# Convergent Use of RhoGAP Toxins by Eukaryotic Parasites and Bacterial Pathogens

**DOI:** 10.1371/journal.ppat.0030203

**Published:** 2007-12-28

**Authors:** Dominique Colinet, Antonin Schmitz, Delphine Depoix, Didier Crochard, Marylène Poirié

**Affiliations:** 1 UMR 1112 UNSA-INRA “Réponses des Organismes aux Stress Environnementaux”, Sophia-Antipolis, France; 2 USM504 “Biologie Fonctionnelle des Protozoaires” - EA3335 Muséum National d'Histoire Naturelle, Paris, France; Stanford University, United States of America

## Abstract

Inactivation of host Rho GTPases is a widespread strategy employed by bacterial pathogens to manipulate mammalian cellular functions and avoid immune defenses. Some bacterial toxins mimic eukaryotic Rho GTPase-activating proteins (GAPs) to inactivate mammalian GTPases, probably as a result of evolutionary convergence. An intriguing question remains whether eukaryotic pathogens or parasites may use endogenous GAPs as immune-suppressive toxins to target the same key genes as bacterial pathogens. Interestingly, a RhoGAP domain–containing protein, LbGAP, was recently characterized from the parasitoid wasp Leptopilina boulardi, and shown to protect parasitoid eggs from the immune response of *Drosophila* host larvae. We demonstrate here that LbGAP has structural characteristics of eukaryotic RhoGAPs but that it acts similarly to bacterial RhoGAP toxins in mammals. First, we show by immunocytochemistry that LbGAP enters *Drosophila* immune cells, plasmatocytes and lamellocytes, and that morphological changes in lamellocytes are correlated with the quantity of LbGAP they contain. Demonstration that LbGAP displays a GAP activity and specifically interacts with the active, GTP-bound form of the two *Drosophila* Rho GTPases Rac1 and Rac2, both required for successful encapsulation of *Leptopilina* eggs, was then achieved using biochemical tests, yeast two-hybrid analysis, and GST pull-down assays. In addition, we show that the overall structure of LbGAP is similar to that of eukaryotic RhoGAP domains, and we identify distinct residues involved in its interaction with Rac GTPases. Altogether, these results show that eukaryotic parasites can use endogenous RhoGAPs as virulence factors and that despite their differences in sequence and structure, eukaryotic and bacterial RhoGAP toxins are similarly used to target the same immune pathways in insects and mammals.

## Author Summary

Recent data have highlighted strong similarities between insect and mammalian immune pathways, raising the question whether they might be similarly targeted by parasites/pathogens. Endoparasitoid wasps are insect mortality agents that perform their development inside their host. They are widely used in biological control, but little is known of the way in which they disable the host immune response. Here, we report the first characterization to our knowledge of a protein–protein interaction between a parasitoid immune-suppressive factor, LbGAP, and its host targets, thus explaining its physiological effects on host immune cells. Strikingly, bacterial toxins containing a domain functionally related to that of LbGAP are known to alter human macrophages by targeting the same molecules. Demonstration that insect and mammalian pathogens/parasites, whether eukaryotic or prokaryotic, can use evolutionary convergent molecules “built” to target the same key factors in immune pathways, represents a new step in understanding the evolution of host–pathogen interactions. In addition to the potential of discovering new bioinsectides, studying virulence strategies of insect parasites might thus help to better characterize vertebrate–pathogen interactions and to assess the role of evolutionary convergence in the evolution of virulence.

## Introduction

Inactivation of host Rho GTPases, which are known to be involved in several cellular processes, including the regulation of the cytoskeletal rearrangements necessary for cell-shape change and migration [1–3], is a widespread strategy employed by bacterial pathogens to manipulate mammalian cellular immunity [4]. ExoS from Pseudomonas aeruginosa, SptP from Salmonella typhimurium or YopE from Yersinia spp. are all virulence factors that mimic eukaryotic Rho GTPase-activating proteins (GAPs), which are important down-regulators of Rho GTPase proteins, to target mammalian GTPases. These bacterial toxins contain a GAP domain with no obvious sequence similarity to eukaryotic proteins and exhibit a different folding, suggesting that they are the product of convergent evolution [4,5]. An intriguing question is then whether this virulence strategy is specific to bacteria or if RhoGAPs are similarly used by eukaryotic parasites or pathogens to target host immunity in addition to their endogenous cellular function.

Endoparasitoid wasps are insects that develop in the body cavity of their host, eventually killing it, and are widely used for biological control. Insect hosts have evolved immune defenses against parasitoids that, if successful, result in the formation of a melanized capsule around the wasp egg and end with the death of the parasitoid [6,7]. In *Drosophila*, plasmatocytes and lamellocytes are the main hemocyte cells responsible for cellular encapsulation [8]. Small, rounded plasmatocytes are the predominant form of hemocytes in non-parasitized larvae, while large, flat lamellocytes are rarely seen in healthy *Drosophila*. Following wasp oviposition, the number of circulating hemocytes and more particularly of lamellocytes increases [9]. Plasmatocytes are first seen attaching to the parasitoid egg and spreading around it. Lamellocytes then adhere to the plasmatocytes to form multiple cell layers [10].

To circumvent host immune defenses, parasitoids have developed different strategies mainly based on the use of virulence factors [11,12]. In particular, parasitoid induction of changes in morphology and adhesion properties of host hemocytes has been repeatedly described [12], as for bacterial pathogens of mammals, but still remains poorly understood. This is at least partly due to the lack of information on the molecular targets of parasitoid toxins in the host. Virulent strains of Leptopilina boulardi, a specialist parasitoid of D. melanogaster, induce changes in the morphology of host lamellocytes [9] and disrupt the encapsulation process. A RhoGAP domain–containing protein (LbGAP, previously known as P4) was recently characterized from parasitoid female venom glands [13]. Interestingly, parasitism-induced changes in host lamellocyte morphology are mimicked by injection of LbGAP alone inside *Drosophila* larvae [14]. These results led us to investigate the function of LbGAP in the disruption of the encapsulation process, to characterize its targets inside the host and to compare LbGAP characteristics with those of bacterial GAP toxins. We first report that LbGAP enters *Drosophila* plasmatocytes and lamellocytes and that the quantity of LbGAP inside lamellocytes correlates with the level of morphological changes of these cells. We then demonstrate that LbGAP displays GAP activity and specifically interacts with the active, GTP-bound form of the two *Drosophila* Rho GTPases, Rac1 and Rac2. In addition, we show that the overall structure of LbGAP is similar to that of eukaryotic RhoGAP domains, and we identify distinct residues involved in its interaction with Rac GTPases. We thus demonstrate that eukaryotic parasites can use endogenous RhoGAPs as virulence factors and that, despite their differences in sequence and structure, eukaryotic and bacterial RhoGAPs are similarly used to target insect and mammalian host immune pathways. This result will help to assess the role of evolutionary convergence in the evolution of virulence.

## Results

### LbGAP Enters *Drosophila* Lamellocytes

**Figure 1:**
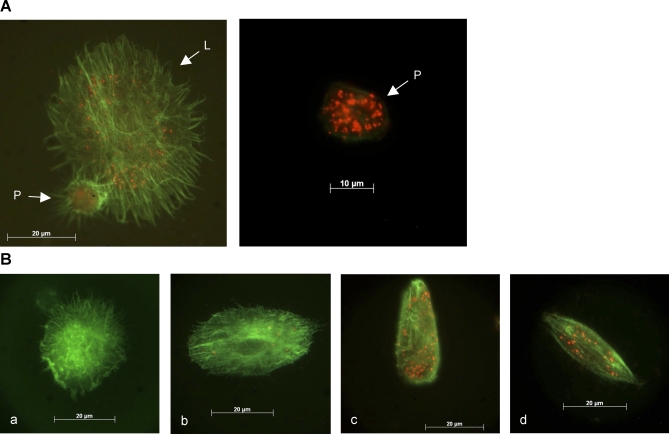
LbGAP Enters *Drosophila* Lamellocytes and Plasmatocytes and Affects Lamellocyte Morphology (A) Example of LbGAP-containing hemocytes. L: lamellocyte; P: plasmatocyte. (B) Classification of lamellocytes into four categories according to their morphological changes. a: Unmodified lamellocyte; b: slightly modified lamellocyte with 1 to 10 LbGAP spots; c: fairly modified lamellocyte with more than 30 LbGAP spots; d: strongly modified lamellocyte with 10 to 30 LbGAP spots. Hemocyte actin cytoskeleton was visualized using phalloidin (green). LbGAP was detected using a specific rabbit polyclonal antibody (red).

To investigate whether LbGAP enters D. melanogaster hemocytes, *Drosophila* larvae were parasitized by L. boulardi females and LbGAP was detected inside hemocyte cells using immunofluorescence. Using LbGAP-specific antibody, we readily detected LbGAP as spots in both plasmatocytes and lamellocytes, whereas no signal was detected in hemocytes from non-parasitized hosts (Figure 1A). LbGAP staining was never localized either at the periphery of the lamellocytes or on the nucleus, thus evidencing that the protein is present inside these very flat cells. Most plasmatocytes displayed a high number of LbGAP spots whatever the time post-infestation. In contrast, the number of LbGAP-stained lamellocytes, as well as the number of lamellocytes containing a high number of spots, significantly increased with the time post-infestation (chi^2^ = 126.26; df = 3; p <0.001 and chi^2^ = 19.9; df = 1; p <0.01, respectively). Spots were detected inside 45% to 50% of lamellocytes 6 hours after parasitism and inside 70% of lamellocytes 48 hours post-infestation. The proportion of modified lamellocytes also increased with the time post-infestation, reaching more than 60% 48 hours following parasitism. Finally, a significant correlation was observed between the level of morphological changes of lamellocytes and the number of LbGAP spots they contain when categorizing cells according to their shape and the number of recorded spots (chi^2^ = 402.92; df = 9; p <0.001). Bipolar modified cells had usually considerable LbGAP staining, whereas most discoidal unmodified lamellocytes did not contain any spot (Figure 1B). These results thus demonstrate the direct involvement of LbGAP in affecting lamellocyte morphology.

### LbGAP Displays a RacGAP Activity

**Figure 2:**
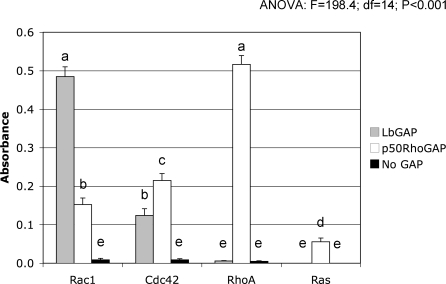
LbGAP Displays GAP Activity *In Vitro* Absorbance at 650 nm is correlated with the amount of Pi released from GTP-bound human Rac1, Cdc42, RhoA or Ras, either in the presence of LbGAP or p50RhoGAP as a control or in the absence of any GAP. Grey bars: GAP activity in the presence of LbGAP. Open bars: GAP activity in the presence of p50RhoGAP. Black bars: GAP activity in the absence of GAP. For each value, bars represent the standard error of three measurements. Different letters above the bars designate significantly different GAP activity results (p <0.001).

RhoGAPs stimulate the low intrinsic GTPase activity of Rho GTPase proteins, leading to conversion of GTP-bound active forms of Rho GTPases to GDP-bound inactive proteins. In order to determine if LbGAP has a GAP function in host cells, we carried out *in vitro* GAP assays using LbGAP protein produced in Escherichia coli. Experiments were performed with human RhoA, Rac1 and Cdc42 Rho GTPases. Human Ras, belonging to the Ras GTPase family, was included as a control. The GAP domain from human p50 RhoGAP, which stimulates GTPase activities of RhoA, Rac1 and Cdc42 *in vitro*, was used as a positive control. Negative controls consisted in the omission of either small G-protein or GAP protein. In these studies, LbGAP significantly increased the GTPase activity of human Rac1 and Cdc42 (F = 198.4; df = 14; p <0.001). It did not, however, activate the conversion of GTP to GDP for RhoA and Ras. The GAP activity towards Rac1 was approximately four times higher than towards Cdc42, suggesting that Rac GTPases are the preferred substrates of LbGAP (Figure 2). However, RhoGAPs that display *in vitro* activity towards multiple Rho proteins sometimes act on a single GTPase *in vivo* [3,15,16], and LbGAP activity towards Rac1 and Cdc42 thus remained to be confirmed using *in vivo* assays.

### LbGAP Physically Interacts with Rac GTPases

**Figure 3:**
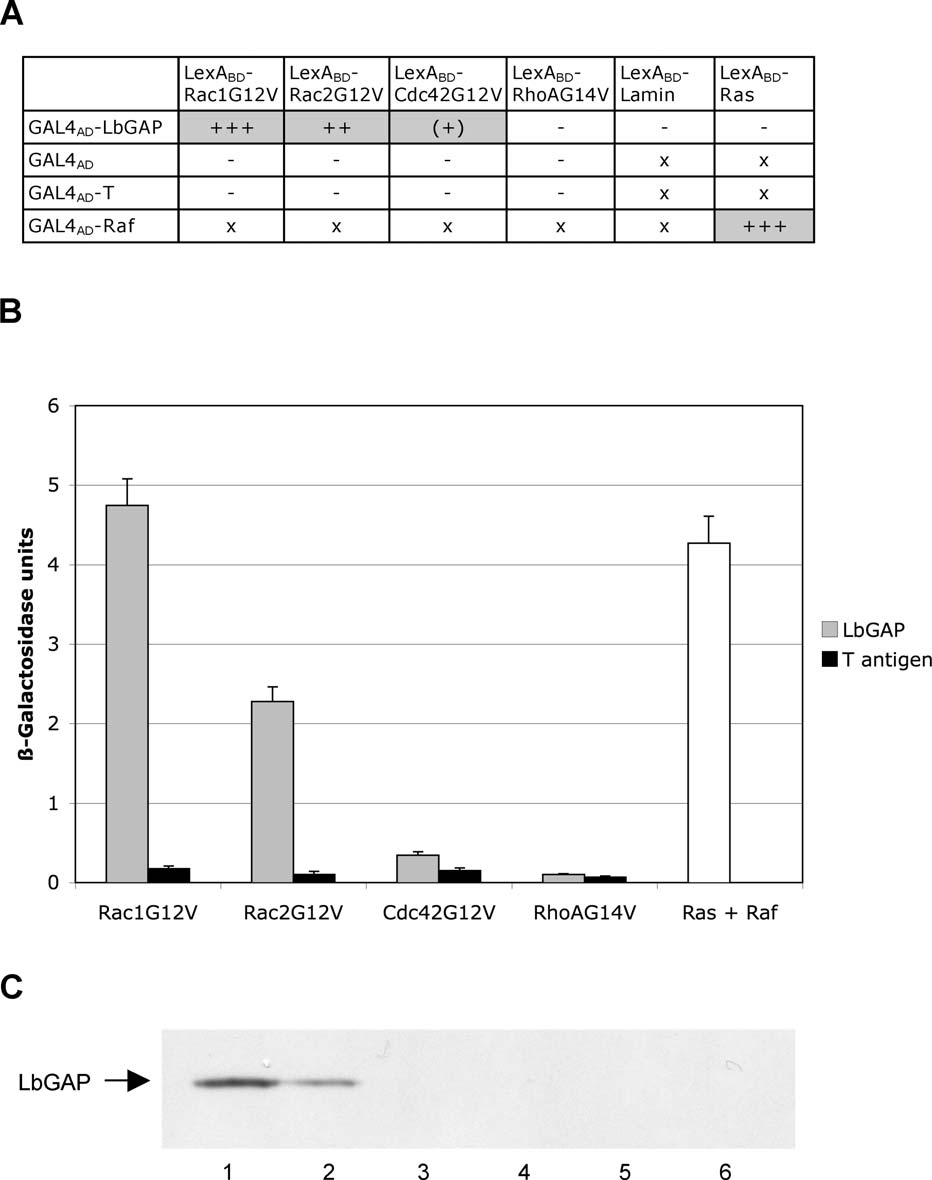
LbGAP Interacts with Rac1 and Rac2 (A) Results based on growth on selective medium lacking histidine and qualitative ß-galactosidase overlay assays. x: non-tested; -: no interaction; (+): very weak interaction; ++: mean interaction; ++++: strong interaction. (B) Interactions with Rac1G12V, Rac2G12V, Cdc42G12V, and RhoAG14V were assayed by measuring ß-galactosidase activity in total protein extracts. Grey bars: ß-galactosidase activity in the presence of LbGAP. Black bars: ß-galactosidase activity in the presence of T antigen. Open bar: Positive control interaction between Ras and Raf. For each value, bars represent the standard error of three measurements. (C) GST pull-down assay. LbGAP protein was synthesized using rabbit reticulocyte lysates and mixed with equal amounts of GST-Rac1G12V or GST proteins bound to Glutathione Sepharose beads. After incubation, the mixtures were subjected to SDS PAGE analysis. 1: LbGAP + GST-Rac1G12V. 2: LbGAP + GST-Rac2G12V. 3: LbGAP + GST. 4: LbGAP alone. 5: GST-Rac1G12V alone. 6: GST-Rac1G12V alone.

Yeast two-hybrid analysis was performed to identify the host targets of LbGAP and to link its GAP activity with physical *in vivo* interactions. In order to stabilize interactions, we used the G12V mutated forms of *Drosophila* Rac1, Rac2 and Cdc42 GTPases and the G14V mutated form of *Drosophila* RhoA [17]. Each of these mutants is deficient in GTPase activity and therefore constitutively blocked in the GTP-bound active conformation [18]. Fusions of the GAL4 activation domain with LbGAP were expressed in yeast together with fusions of the LexA-DNA binding domain with either Rac1G12V, Rac2G12V, Cdc42G12V or RhoG14V. Direct *in vivo* interaction of LbGAP with small GTPases was measured as the ability of transformed yeast to activate the transcription of *HIS3* and *lacZ* reporter genes, both under the control of the LexA-binding sequences. Yeast growth on a selective medium lacking histidine together with qualitative detection of ß-galactosidase activity revealed that LbGAP strongly interacts with Rac1G12V and Rac2G12V (Figure 3A). By contrast, LbGAP showed only very weak interaction with Cdc42G12V and no interaction with RhoAG14V.

The strength and specificity of the interaction between LbGAP and Rac GTPases was then estimated by titration of ß-galactosidase activity (Figure 3B). Substantial activity was obtained using coexpression of GAL4AD-LbGAP and either LexABD-Rac1G12V or LexABD-Rac2G12V but not in combination with non-specific sequences. The ß-galactosidase activity resulting from the interaction between LbGAP and Rac1G12V was similar to that obtained for the positive control (pLex-Ras + pGAD-Raf), supporting the idea that this interaction is strong and specific. The ß-galactosidase activity was approximately two times higher than that obtained for the interaction between LbGAP and Rac2G12V, suggesting that LbGAP interacts more strongly with Rac1G12V than with Rac2G12V.

To further validate the yeast two-hybrid approach, we performed a GST pull-down assay using LbGAP synthesized *in vitro* in rabbit reticulocyte lysates and Rac1G12V and Rac2G12V expressed in bacteria as a fusion with GST. LbGAP was pulled down by Rac1G12V and Rac2G12V but not by GST alone, thereby demonstrating the specificity of the interaction between LbGAP and Rac GTPases (Figure 3C).

### LbGAP Interacts with the Active Form of Rac GTPases

To determine whether activation of the GTPases influences binding to LbGAP, a second constitutively activated variant (Q61L) and the GDP-bound inactive form (T17N) of Rac1 were used in two-hybrid assays. The leucine substitution in Rac1Q61L results in a strongly decreased GTPase activity, as in Rac1G12V, hence the mutant is always in the active, GTP-bound state [19]. On the contrary, the dominant negative Rac1T17N mutant has a severely reduced affinity for GTP and is blocked in the inactive GDP-bound conformation [18]. Since Rac1 and Rac2 shares more than 90% sequence identity with absolute conservation of mutagenized residues, the following experiments were performed with Rac1.

**Figure 4:**
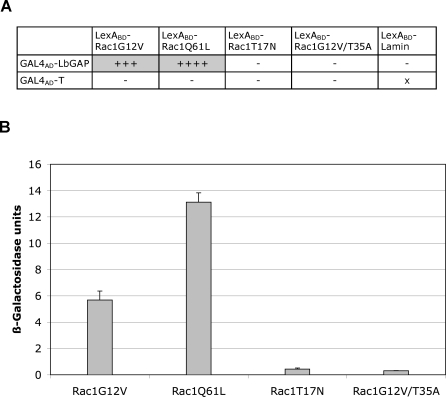
LbGAP Specifically Interacts with the Active, GTP-Bound Form of Rac GTPases (A) Summary of results obtained for LbGAP interaction with Rac1G12V, Rac1Q61L, Rac1T17N, and Rac1G12V/T35A by growth on selective medium lacking histidine and qualitative ß-galactosidase overlay assays. x: non-tested; - : no interaction; +++, ++++: strong interaction. (B) Interactions with Rac1G12V, Rac1Q61L, Rac1T17N, and Rac1G12V/T35A assayed by measuring ß-galactosidase activity in total protein extracts. Grey bars: ß-galactosidase activity in the presence of LbGAP. For each value, bars represent the standard error of three measurements.

Only the coexpression of LbGAP with Rac1G12V and Rac1Q61L allowed yeast to grow on a selective medium without histidine and activate the *lacZ* reporter gene (Figure 4A and 4B). There was no detectable interaction between LbGAP and Rac1T17N (Figure 4A and 4B). We finally used the T35A mutation which affects the effector loop known to be essential for interaction of GTP-bound Rho GTPases with specific effectors, including RacGAP proteins [19,20]. As expected, the mutant form Rac1G12V/T35A was not able to interact with LbGAP (Figure 4A and 4B). These results demonstrate that only the active, GTP-bound form of Rac GTPases binds to LbGAP.

### Structural and Mechanistic Similarities

**Figure 5:**
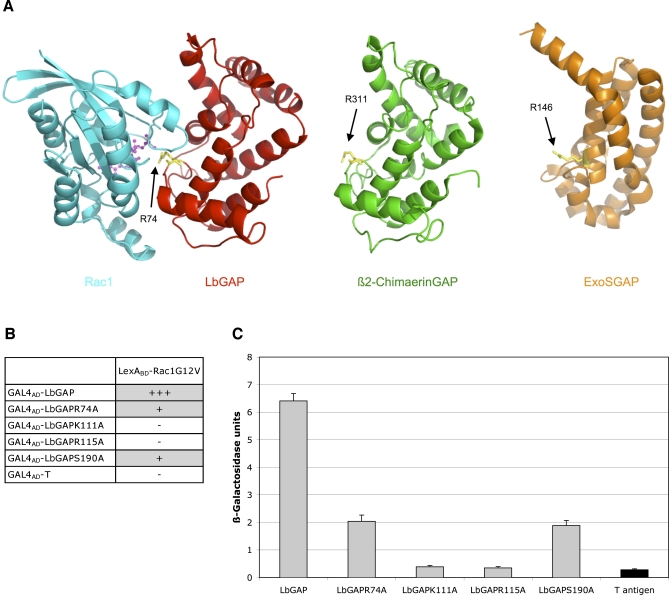
Molecular Modeling of LbGAP and *In Vitro* Mutagenesis (A) Tertiary structure of the Rac1-LbGAP complex and comparison of LbGAP structure with ß2-ChimaerinGAP and ExoSGAP folds. The LbGAP structure is colored red, the ß2-ChimaerinGAP structure is colored green, the ExoSGAP structure is colored orange, and the Rac1 structure is colored blue. Helices are shown as ribbons. Protruding LbGAP Arg74, ß2-ChimaerinGAP Arg311, and ExoSGAP Arg146 are shown as sticks and colored yellow. GDP and AlF3 are shown as ball-and-sticks models and colored in magenta. (B) Summary of results obtained for Rac1G12V interaction with LbGAPR74A, LbGAPK111A, LbGAPR115A, and LbGAPS190A mutants by growth on selective medium lacking histidine and qualitative ß-galactosidase overlay assays. -: no interaction; +: weak interaction; +++: strong interaction. (C) Interactions with LbGAPR74A, LbGAPK111A, LbGAPR115A, and LbGAPS190A mutants assayed by measuring ß-galactosidase activity in total protein extracts. Grey bars: ß-galactosidase activity in the presence of LbGAP. For each value, bars represent the standard error of all three measurements.

Using the Swiss Model First Approach Mode and the similarities with structurally characterized RhoGAP domains as support, we constructed a 3D model for LbGAP. The predicted structure is similar to that of the eukaryotic RhoGAP fold [21–24] and has very few differences with that of the RhoGAP domain of ß2-chimaerin (Figure 5A), a Rac-specific GTPase-activating protein [22]. As expected, there is no obvious structural homology with the GAP domain of P. aeruginosa Exoenzyme S (ExoSGAP) major toxin (Figure 5A), whose tertiary fold was previously shown to differ from that of eukaryotic RhoGAPs [25]. Superimposition of structures allowed us to model the structure of Rac and LbGAP complexed with the transition-state analogue GDP.AlF3. The modeled complex shows Arg74 of LbGAP forming an arginine finger that is introduced within the active site of the GTPase in the vicinity of Rac Gln61 and GDP.AlF3 (Figure 5A). This arginine residue is conserved in all GAP proteins. In the GAP-GTPase complex, it stabilizes the GTPase invariant glutamine residue 61 or 63 to facilitate the catalysis of GTP to GDP [21].

Further analysis of the LbGAP structure suggested that the exposed residues Lys111, Arg115 and Ser190 might also participate directly in the interaction with Rac GTPases. Lys111 and Arg115 residues are conserved among eukaryotic RhoGAP proteins and have been proposed or shown to be involved in binding of Rho GTPases [21,26]. On the other hand, Asn or Thr is usually found in place of Ser190 [21–24,26]. We generated four site-specific mutants of LbGAP, R74A, K111A, R115A and S190A, and compared their binding capabilities to Rac1 with that of wild-type LbGAP. Two-hybrid analysis revealed that the R74A and S190A mutants were still able to interact with Rac1, although the interaction was approximately three times lower compared to that with the wild-type protein (Figure 5B and 5C). In contrast, there was no detectable interaction between K111A and R115A mutants of LbGAP and Rac1. These results indicate that Arg74 and S190 contribute to, but are not essential for, binding to Rac GTPases, while Lys111 and Arg115 seem to be crucial for the interaction.

## Discussion

Adhesion and cell shape changes are an essential part of the insect cellular response against endoparasitoids, and consequently, most of them alter hemocyte morphology or inhibit hemocyte spreading [12,27–29]. Here, we link the physiological effects of a parasitoid virulence factor on host hemocytes with its molecular function and its protein targets in the host.

LbGAP is, to our knowledge, the only GAP domain–containing protein described as a eukaryotic virulence factor involved in immune suppression of host defenses. Our results, together with sequence similarities [13], characterize this toxin as a Rac-specific GAP that specifically targets *Drosophila* Rac1 and Rac2, two GTPases that are more than 90% identical in sequence. LbGAP seems to interact more strongly with Rac1, the significance of which remains to be assessed. Specific targeting of Rac1 and Rac2 by a parasitoid toxin highlights the key role of these GTPases in the regulation of *Drosophila* immune defenses, a role that Rac GTPases also play in mammalian anti-bacterial innate defenses.

Rac GTPases regulate cytoskeletal rearrangements and adhesions necessary for cell-shape change and migration [30]. Interestingly, Rac1 and Rac2 were recently demonstrated to be non-redundantly required for successful encapsulation of L. boulardi eggs [31,32]. Rac1 is involved in hemocyte number increase and induction of lamellocyte formation following infestation, as well as in hemocyte activation and regulation of cellular adhesions in lamellocytes [32,33]. Rac2 has a specific role in hemocyte spreading following attachment to the parasitoid egg and cell junction formation during the encapsulation process [31]. Accordingly, we report that LbGAP enters *Drosophila* hemocytes, plasmatocytes and lamellocytes, and is directly involved in affecting the morphology of circulating lamellocytes.

The question of the mode of entry of parasitoid-derived extracellular toxins in host hemocytes has rarely been addressed. In the CrV1 polydnavirus-encoded protein, a coiled-coil domain containing a putative leucine zipper seems to be required for binding and uptake by hemocytes [34], but there is no such coiled-coil region in the LbGAP sequence. Association of LbGAP with virus-like particles (VLPs), which are present in L. boulardi female venom glands [35], might facilitate its entry in host hemocytes, but still remains to be tested experimentally. This idea is supported both by observation of LbGAP staining as “large spots” inside the hemocytes and by data on L. heterotoma VLPs [36]. In this parasitoid, VLPs, that somehow affect lamellocyte morphology, were described free in the cytoplasm of lamellocytes but restricted to phagocytic vesicles of plasmatocytes [36].

LbGAP has all characteristics of a RhoGAP toxin. However, in contrast to bacterial GAP toxins, it shares broad structural and mechanistic similarities with eukaryotic RhoGAPs and probably corresponds to an endogenous RhoGAP protein. Bacterial GAP toxins lead to the disruption of actin filaments in mammalian host cells and inhibition of bacterial uptake [37]. Interestingly, expression of ExoSGAP was also shown to inhibit Rac GTPase–dependent signalling in *Drosophila* and alter its anti-bacterial immune defense [38].

An open area of research is now to assess the importance of RhoGAP molecules as virulence factors in parasitoid wasps and other eukaryotic parasite/pathogens. Interestingly, a RhoGAP domain–containing protein, VLP2, has been described in another VLP-bearing parasitoid species, Venturia canescens [39], but its involvement as a virulence factor remains to be tested. Other regulators of Rho GTPase function, such as GDP/GTP exchange factors (GEFs), are used by bacteria to circumvent host immunity [4], and might also be used as eukaryotic toxins. As a whole, our data suggest that considering well-studied prokaryotic toxins might help deciphering virulence of eukaryotic parasites.

Research in the last ten years has provided evidence of high similarities between the *Drosophila* immune response and mammalian innate immunity [40,41] and has highlighted the interest of *Drosophila* as a model system for studying the evolution of haematopoiesis [42]. Recent data suggest that insect and mammalian parasite/pathogens might alter similar host immune pathways by injecting functionally related toxins [43,44]. We establish here that these functionally related toxins can also target the same key molecules. In addition to the potential of discovering new bioinsectides, studying virulence strategies of insect parasites might thus help to better characterize vertebrate–pathogen interactions and to assess the role of evolutionary convergence in the evolution of virulence.

## Materials and Methods

### Origin of strains.

The origin of the Leptopilina boulardi Ism strain (Gif stock number 431), virulent on Drosophila melanogaster, has been previously described [45]. The D. melanogaster hop^Tum-l^ strain [46], a tumour-forming stock, was provided by Dr. Hsiling Chiu (City College of City University of New York). Its genotype is y v hop^Tum-l^ on chromosome X. Larvae homozygous or hemizygous for the mutation, identified by their yellow mouth hooks, were used in the experiments since they produce an overabundance of lamellocytes.

### Immunocytochemistry.

Groups of 30 Hop^Tum-l^ larvae were parasitized by five L. boulardi females for 4 hours. The hemocytes were bled from larvae 6, 15, 24 or 48 hours post-parasitism and allowed to attach to a glass slide for 1 hour. The cells were fixed for 10 min with 4% paraformaldehyde, washed in PBS, permeabilized in 0.1% Triton and blocked in 2% BSA. The samples were first incubated with the rabbit polyclonal anti-LbGAP [13] antibody and then with a secondary anti-rabbit antibody conjugated with FluoProbe 494 (Interchim) together with Phalloidin-X5-FluoProbe 505 (Interchim) to visualize F-actin. The slides were mounted using fluorescent mount medium (DakoCytomation). Observations were performed under fluorescence microscopy (Axioplan 2, Zeiss) and digital pictures were taken under 100× oil objective with an AxioCam Zeiss camera controlled by the Axiovision 4 software (Zeiss).

Experiments were performed in triplicate for each post-parasitism time condition and lamellocytes and plasmatocytes were observed for LbGAP and F-actin staining. Lamellocytes were classified into four categories according to their shape (unmodified, slightly modified, fairly modified or strongly modified) and the number of recorded LbGAP spots (no spots, 1 to 10 spots, 11 to 30 spots, more than 30 spots). Three hundred lamellocytes were observed and counted for each sample.

Statistical analysis was performed using the SAS software. The three variables, “Lamellocyte morphology”, “Lamellocyte staining”, and “Time post-infestation”, were entered as categorical variables. Relationships between these variables were then analyzed using multiple logistic regression categorical data modeling (CATMOD), with parameters derived using the maximum likelihood (ML) method. Data were analysed in order to test independence between “Lamellocyte morphology” or “Lamellocyte staining” and “Time post-infestation” and between “Lamellocyte staining” and “Lamellocyte morphology”.

### In vitro GAP assays.

LbGAP was produced as a fusion to GST and then released through cleavage with the Xa factor as previously reported [13]. In vitro GAP assays were performed in triplicates using the RhoGAP Assay Biochem Kit from Cytoskeleton. Occurrence of treatment-dependent differences in GAP activities was tested using ANOVA analyses, followed by pairwise comparisons using Fisher's Least Significant Difference (LSD) test, and the Systat software package.

### Yeast two-hybrid analysis.

The LbGAP cDNA was amplified by RT-PCR using mRNA from L. boulardi female long gland extracts and inserted into the pGADT7 vector by homologous recombination in yeast strain JD53 (MATa, his3–200, leu2–3112, lys2–801, trp1–63, ura3–52). The LexA DNA binding domain plasmids expressing mutated forms of Rho GTPases were kindly provided by Dr. Fauvarque (Département de Biologie Moléculaire et Structurale, CEA Grenoble) and transformed in yeast strain L40 (MATα, trp1, leu2, ade2, GAL4, lexAops-HIS3, lexAops-lacZ) using Gietz protocol [47].

Pairs of interactions were examined individually by mating JD53 and L40 yeast colonies according to the Yeast Protocols Handbook PT3024–1 (Clontech Laboratories). The plasmids expressing Rho GTPases were tested against pGADT7 empty vector and pGADT7-T control vector, encoding a fusion between the GAL4 activation domain and SV40 large T-antigen. Reciprocally, the plasmid producing LbGAP was tested against pLex-Ras and pLex-Lamin control vectors. Interaction between pGAD-Raf and pLex-Ras was used as a positive control.

Interactions were tested by spotting five-fold serial dilutions of cells on minimal medium lacking histidine and supplemented with 3-amino-triazole at 0.5 mM to reduce the number of false positives. ß-galactosidase activity was then revealed on plates as previously described [48]. Finally, quantification of ß-galactosidase activity in liquid assays was performed according to the Yeast Protocols Handbook PT3024–1 (Clontech Laboratories).

### GST pull-down assay.

The production of the GST-Rac1G12V and the GST-Rac2G12V fusion proteins and GST alone was performed according to the GST Gene Fusion System Handbook (Amersham Biosciences). For the pull-down assay, equal amounts of GST-Rac1G12V, GST-Rac2G12V or GST alone were added to 10 μl of rabbit reticulocyte lysates (TNT Quick Coupled Transcription/Translation Systems, Promega) programmed with pGADT7-LbGAP in the presence of TranscendTM Biotin-Lysil-tRNA (Promega). After incubation overnight with 20 μl glutathione-Sepharose, proteins bound to the Sepharose beads were washed, resolved by SDS-PAGE and electroblotted to an Immobilon-P Transfer Membrane (Millipore). Biotinylated proteins were visualized using the TranscendTM Non-Radioactive Translation Detection System (Promega).

### Molecular modeling of LbGAP.

The RhoGAP domain of LbGAP was modeled by homology using Swiss Model [49–51] with human ß2-chimaerin [22] and chicken Graf [23] structures as templates. The model quality was assessed by Whatcheck [52] and Procheck [53]. The structure of Rac and LbGAP complexed with the transition-state analogue GDP.AlF3 was modeled by superimposing the RhoGAP domain of the p50RhoGAP/RhoA-GDP.AlF4 complex [21] on LbGAP. The structure of Rac-GDP.AlF3 as observed in complex with ExoSGAP [25] was then superimposed on the RhoA portion of the p50RhoGAP/RhoA-GDP.AlF4- structure. The molecular structures were visualized and superimposed using PyMol (http://pymol.sourceforge.net/).

### In vitro mutagenesis.

The R74A, K111A, R115A and S190A mutations were introduced into the LbGAP cDNA using the QuickChange XL Site-Directed Mutagenesis Kit (Stratagene).

## Supporting Information

### Accession Numbers

GenBank (http://www.ncbi.nlm.nih.gov/Genbank/index.html) accession numbers for the genes and proteins mentioned in the text are: Leptopilina boulardi LbGAP (previously known as P4) (CAI28919), *Drosophila* Rac1 (NP_476950), *Drosophila* Rac2 (NP_648121), *Drosophila* Cdc42 (AAD43787), *Drosophila* RhoA (NP_477098), Pseudomonas aeruginosa ExoSGAP (1HE1).
